# Lectures on Scarlet Fever

**Published:** 1851-09

**Authors:** Caspar Morris

**Affiliations:** Late Lecturer on Practical Medicine in the Philadelphia Medical Institute


### MEDICAL EXAMINER,

AND

### RECORD OF MEDICAL SCIENCE.

NEW SERIES.—NO. LXXXI.—SEPTEMBER, 1851.

---

### ORIGINAL COMMUNICATIONS.

---

*Lectures on Scarlet Fever.* By CASPAR MORRIS, M. D., late Lecturer on Practical Medicine in the Philadelphia Medical Institute.

### LECTURE VII.

While the general character of the *simple* form of Scarlet Fever is sthenic, and, as I have remarked when describing the treatment appropriate to it, will bear depletion better than the other varieties, though never demanding it, the *anginose form* is not so uniform in this respect. In different epidemics it presents every grade of action, from one of acute inflammatory fever, till it is merged, by imperceptible shades, into the low or malignant form. Hence arises the great variety in the modes of treatment recommended by different authors, all equally entitled to your confidence; and the great difference in the mortality in different years, or in the same year at different places. This difference in mortality cannot always be ascribed to different modes of treatment, though this does undoubtedly exert a powerful influence. In the London Fever Hospital it varied, in the course of eleven consecutive years, from *one* fatal case in every

*six*, to *one* in *forty;* and an equally curious variation will mark the practice of most medical men, not only in the course of years, but even during the same year, in different parts of the same town, or district of country, and in different families. The observation of these facts has caused me to be very cautious in the expression of condemnation of any particular mode of practice. The candid remark of the judicious and upright Dr. Fothergill may be borne in mind with great advantage: "In some cases the disease appears to be of so mild a nature, and so benign, as to require but little assistance from art; persons even recover from it under the disadvantages of unskilful and injurious management; whilst in others the progress of the symptoms is so rapid, and the tendency to corruption so strong, that nothing seems able to oppose it." It is not, therefore, just, always to estimate the advantage of a plan of treatment by the recovery of a limited number of patients. It may merely indicate that the power of nature for self-preservation is sufficiently strong to resist the combined injurious influences of the disease and the treatment. I desire to make this remark with entire candor, and that it should be applied with equal fairness to the treatment I myself suggest, as to that equally strongly recommended by others; while, at the same time, I would assure you that it is not without personal experience of the effect of other plans, that I have adopted my own.

This same general remark as to the limited duration of the original disease, the importance of which I endeavored to impress upon you when the simple form was under consideration, is equally applicable to this. It is of far greater importance, however, to keep it ever in your mind, and to allow it to modify your practice. The greater urgency of the symptoms appears to demand a proportionate increase of the energy of treatment. The local lesions are frequently so severe from the very onset, as to require anxious care; and the influence they exert in prolonging the fever, by their irritation after the primary disease has subsided, is well calculated to render the treatment obscure, and the result doubtful. Few minds are so well balanced as to be able to resist the influence of these circumstances, or capable of such nice discrimination as will enable them to arrange the symptoms and adopt the treatment without improper bias. Not only does the severity of the local lesions, in many cases, incite to the adoption of a treatment needlessly active, but the fear of the result of these local inflammations is often so great, as to overwhelm the judgment. I have known not a few lives sacrificed to the anxiety to avert anticipated evils, and to cure a condition which art can only *relieve*, while nature herself is working the cure, in the regular course of events. Nor have I any wish to screen myself from the censure of being a participant in such results. The remembrance of too many cases in which I have had reason to regret a too energetic treatment, whether depletory or stimulating, urges me thus to press upon your attention, opinions which have been formed at the expense of much personal observation.

The occurrence of a local lesion giving rise to the *anginose* variety of Scarlet Fever, does not of itself involve any change in the general principles of treatment already laid before you. In its slightest degree the anginose manifests itself by a slight tenderness about the angle of the jaw, with some enlargement of the lymphatic glands. If the condition of the fauces is examined, the membrane lining the arches and covering the tonsils and uvula, will be found more intensely red, and sometimes slightly œdematous. From this condition the severity of the local affection gradually increases, till it assumes the various appearances formerly described.

The only change in the treatment demanded by this local affection, when there is no tendency to sloughing of the inflamed organs, nor general disposition to prostration of the vital force, is attention to those agents which will afford present relief from the discomfort it occasions. The external swelling and tenderness will yield to the soothing influence of warmth and moisture. When properly applied, this is always grateful, but when done in a slovenly or careless manner, is exceedingly offensive. A flannel cloth wrung out of hot water, and applied with moderate firmness, and of several folds thickness, and then covered with a layer of oiled silk, and that enveloped in a nice napkin, will retain the necessary degree of warmth and moisture a long time. It is lighter than the poultices commonly resorted to, and is free from the smell which renders them offensive, and does not leave the dried deposit which forms around the edges of the cataplasms.

Next to this in adaptation is a thin layer of Indian mush, thoroughly boiled till it forms an adhesive mass, and applied in a thin flannel envelope, taking the same precautions to avoid evaporation. You may think these are matters which may be left to the attention of the nurse; but you will find that not only your own reputation will be increased, by your ability to give minute directions about apparently trifling matters; but, what is of vastly greater consequence, the present comfort of your patients will be increased, and these little things influence, in a great degree, the ultimate result. To no portion of human experience is the line of the satirist more applicable, than to that which comes under the notice of the medical adviser:

> "Let school bred pride dissemble all it can,
> These *little things* are *great* to little man."

Great not only in relation to comfort, but to results.

But while warmth and moisture externally applied are thus important, the internal condition requires an opposite treatment. There is nothing so effectually soothes the internal irritation, and averts the probable results of the excited action of the capillaries of the mucous membrane, as the free use of *ice.* In the same manner as the sponging or ablution of the surface, it acts not only in the relief of the local inflammation but also on the general nervous system. The natural instinct of the patient calls for such treatment, and its appropriateness will commend it to every one ready to throw off the trammels of authority. I had long employed it to great advantage before I was aware that it had been used by any other person, but am happy to be able to fortify my testimony to its usefulness, by the report of Dr. S. Jackson, of Northumberland, which is given in the North American Medical and Surgical Journal. Dr. Jackson deserves, I believe, the credit of first drawing the attention of the profession to its use, through the agency of the press. I direct the ice to be divided into *small* fragments, such as can be easily held in the mouth without dissolving. When thus prepared, if wrapped well in flannel and placed in a bowl at the bedside, so as to be easily accessible, the patient may use it as freely as agreeable. When old enough to perform the act of gargling, iced water is the best article for that purpose. In a large majority of cases I have found this local treatment, joined to the same general course previously recommended, lead to the entire subsidence of the local symptoms simultaneously with the decline of the primary fever.

Would that I could promise so happy a result in every case, even where the primary symptoms are least threatening. It occasionally happens that ulceration of some part of the mucous membrane occurs, either in the posterior nares or the fauces, and gives rise to a profuse secretion of viscid mucus, or a discharge of an acrid fluid, which spreads the destruction over adjacent parts, and, as I have mentioned already, even excoriates the skin when it is allowed to rest upon it. It is almost equally important to relieve both of these conditions. The latter, by spreading the ulcerative process, adds to and prolongs the irritative fever, while the former very materially increases the risk of a fatal termination by the serious impediment to free respiration which it interposes. The vital powers already prostrated by the depressing agency of the miasm which produces the disease, the blood poisoned by its deleterious influence, the life or death of the patient may depend on the freedom with which air is drawn into the lungs. The nervous force diminished and the muscular power abated, we have only to obstruct the air passages through which the life-giving oxygen is admitted, to cut off the only avenue for hope. How often have I seen a poor child lie with the nostrils—the natural breathing channel—entirely obstructed, the mouth unnaturally open to supply a supplementary avenue for air, yet tossing and struggling in instinctive efforts for fresh air to vivify the blood; while the injected conjunctiva, the upturned eye, and the livid hue, all told how vain was the effort. Though when it has reached this point in the downward course, little hope can cheer our efforts, even then they should not be abandoned, as relief to suffering is within our reach, even though death may finally conquer. Gargling is wholly useless; the seat of the diseased action is beyond its reach, and the barbarous substitute of swabbing is but little more effective, with all the pain it gives to the patient and trouble to the operator. The *syringe* affords a most effectual and very easily applied remedy. When no other was accessible, I have employed a common gill pewter pipe; but the neat glass syringe now used for cleansing the ear is admirably adapted for this service. Water alone is not sufficient to inject. A little nice soap, which has no volatile oil in it, may be diffused in the water, in those cases where the irritation of the mucous membrane gives rise to a viscid tenacious secretion; or if this is not effectual in changing the action, a very *weak* solution of sulphate of copper or zinc. Either of these agents coagulates the mucus and facilitates its removal at the same time that it acts kindly on the diseased membrane. In those cases in which the secretion is thin and acrid, I have found simple lime water the best detergent. They should be thrown into the nostril, care being taken to have the head elevated at the time and the person of the child inclined forward, while the direction given to the syringe should be such as will cause the fluid to pass toward the posterior nares, and not toward the cells and sinuses. When the throat is much swollen, the fluid thrown into one nostril will often return through the other, thus effectually cleansing both; in other cases it will be rejected through the mouth. The greatest trouble it causes is some little retching or sneezing, either of which is advantageous to the patient. I have often saved life by this simple resort, and commend it to your notice as of great practical value. The solution of sulphate of copper or zinc should not be employed during the first five days of the disease. They then only increase the irritation and aggravate the suffering of the patient, as well as promote the ulceration they are so adapted to cure in the later stage.

The diet of the patient in anginose scarlet fever should also claim the attention of the physiciam. During the earlier stage, the remarks made on the simple form are equally applicable here. Water ices, iced cream or chocolate ice, subacid fruits and whey, either frozen or dissolved, are excellent, if used with moderation. As the primary fever subsides, animal broths, and bread and milk are well adapted; still keeping in view the fact that quantity is quite as important as quality. Purging as a curative agent is needless, though it is important to secure a regular action of the bowels, which may be readily effected either by small quantities of calcined magnesia or the use of the neutral mixture. We meet with some cases in which there is a tendency to diarrhœa, caused by the irritation of the acid secretions swallowed by the child. These are most effectually treated by the magnesia in very small doses, which absorbs the acid and carries off the cause of irritation. It should be followed by suitable doses of Dover's powder or laudanum.

But whilst I thus recommend, as the result of my own observations, in a field by no means limited, a treatment so simple, and by some thought inert, when brought into contest with a disease of so formidable character, I am bound to inform you that there are not wanting many authors, and those, too, of the very highest authority in medical science, who urge a very different plan. General bleeding is not without its advocates, as well among our own medical authors as those of Europe. Even those writers who most recommend its employment, do not hesitate to admit that there are epidemics to which it is inappropriate. Cerebral complications have been thought especially to demand it; on that point I have already delivered my opinion. I have never known a patient to recover from this form of scarlet fever when I employed the lancet. The rapidity of the circulation, the heat of the surface, and the buffy coat on the blood, which have been adduced as indications for bleeding or justification of its use, have all been proved by more accurate observation to be susceptible of a different construction. I do not mean to assert that it is not possible for the disease to assume a character which may demand such treatment. I know it to be capable of changes so extreme, that I can easily believe such may have been its character without my having met with the cases. But even many of those authors who coincide with me in the abandonment of general bleeding, still urge the necessity of the topical abstraction of blood by cups or leeches. This practice is, I think, open to still greater reprehension than the other. Not only is it subject to the same objection on account of its depressing influence, but there is superadded the liability to ulceration of the bites of the leeches or the gashes of the scarificator, which give rise to tedious sores, and often leave unsightly scars, even if the vigor of the patient be sufficient to carry him through the combined injurious influences of the disease and the treatment. But while modern observation has cast a shade of doubt on the necessity for bloodletting in even the pure phlegmasiæ, it has brought still greater discredit on the resort to it in the mixed cases of local inflammation with specific fever. Even quinsy, which is more closely allied to anginose scarlet fever than any other disease, is rarely, if ever, arrested in its progress by either general or topical bloodletting. While, therefore, there is much to discountenance the resort to these measures, there is, on the other hand, little to invite to their use.

The employment of emetics has been highly commended by many authors, and their use may be vindicated more easily than that of bleeding. The shock given to the nervous system is by some thought to promote the development of the disease. This is at least a questionable assertion. I believe the only mode in which they can act advantageously, is by the discharge of the contents of the stomach, which might, if retained, become sources of irritation, and by the production of that determination to the surface which accompanies the act of vomiting. Antimonial emetics are wholly unsuited. Ipecacuanha is the best article that can be employed; and next to it I should rank the powdered alum, so highly and justly recommended by Dr. Meigs in croup. Purging, beyond the mere evacuation of the bowels, is not only uncalled for, but pernicious. It cannot remove the specific irritation of the mucous membrane of the alimentary canal, and adds another to that already existing. Even Dr. Hamilton, while he lauds highly the advantages of a free purge in the beginning, admits that it is not required subsequently. The recovery of patients after vomiting or purging, cannot be adduced as evidence of the benefit of the practice, for reasons already assigned; and when there are any signs of irritation of the mucous membrane, they are decidedly injurious. Dr. Fothergill, speaking of the treatment of the disease, says: "Bleeding was prejudicial," but with that caution which gives the more weight to his authority, adds, "in general; some admit of it at the first attack, but later it never fails to aggravate the symptoms, and in some cases it appears to have produced fatal consequences. Nor has purging," he continues, "been observed to be more beneficial, and nitrous cooling medicines produce the like effect." Gentle emetics, if administered early, he commends highly, declaring that they often cure the disease, even when the symptoms are most threatening.

It not unfrequently happens that at the close of the first week of the disease, as the primary fever subsides, the pulse loses its frequency, the skin becomes cool and moist, and the patient languid, with loss of appetite. A very moderate use of quinine in such circumstances is useful. Great care is requisite not to push it too far, either in quantity or the length of time. A few days bring you to the period which is especially liable to inflammatory sequelæ. A return of the frequency of the pulse, and an increase of the languor, will mark the occurrence of this condition, and should cause an immediate suspension of the tonic treatment, and great care about the diet. This is especially necessary, as it is impossible to foresee in which of the organs liable to the secondary affections the mischief may develop itself; and the attacks are so formidable, that it is important to guard against the very beginning of the evil. A cerebral or cardiac inflammation would be materially aggravated by the influence of the quinia. The condition of the urine is also an important means of determining how far it may be proper to pursue the tonic treatment. While that secretion continues abundant and clear, and the skin cool and perspirable, there can be no risk. The interruption of the secretion from either of these eliminating organs, or the deep color or turbidity of the urine, would at once indicate the propriety of suspending the quinine.

It is in these cases that we frequently meet with that very formidable complication, inflammation of the larynx, producing all the symptoms of croup. Even in these cases no benefit will result from depletion, or any other depressing agent; dependent on the extension of the specific inflammation from the fauces, it must be relieved by local treatment alone. Emetics of alum or ipecacuanha are the most valuable of our remedies, and may be repeated as often as the urgency of the symptoms requires; care being taken at the same time to afford proper support, by suitable nourishment and stimulants. One of the happiest results I ever achieved, was in the recovery of a child of about two years of age, in whose case the disease was ushered in by convulsions, which yielded to tepid baths, with cold affusions to the head. The disease then ran its regular course, accompanied with much sloughing of the fauces and uvula, and leaving an exceedingly red ulcer. This I treated with the injections recommended above, and was flattering myself with the expectation of a speedy recovery, when the harsh clangor of a croupy cough sounded like the knell of hope. I procured at once a stick of nitrate of silver, fastened in a quill, and, depressing the tongue, thrust it deep into the fauces, with the design of extending the application to the very verge of the glottis itself. A sudden struggle of the child broke off the caustic from the quill, and, to my indescribable horror, one gulp carried into the stomach of the child about two drachms of the caustic. To fail in one's efforts to cure is disheartening enough,—to feel that those efforts, however well meant, have rendered certain the triumph of death, is like being dragged at the chariot wheel to swell the triumph. Determined not to yield without an effort, and saying nothing of the accident, I called for the salt cellar, which was promptly handed me, and compelled the child to take large and repeated draughts of a strong infusion of the chloride of sodium. This of course produced free emesis, at the same time that it rendered insoluble and inert the poisonous mass thus undesignedly passed into the stomach. Whether the result may be ascribed to the action of the caustic, or that of the saline application to the local disease, or, what is more probable, to the oft repeated vomiting, I am not prepared positively to assert. I am disposed to divide the honor between the two latter influences, providentially, though unintentionally applied. Certain it is that most unexpectedly the child recovered.

But the most judiciously planned treatment, though faithfully carried out, will not ensure a successful result, even in those cases which do not manifest any very unfavorable symptoms at the beginning. The impeded deglutition, the labored respiration, the swollen lymphatics, occurring about the end of the first week, or even sooner, all speak of a condition full of danger, while a hard, quick pulse, which generally accompanies this condition, would appear to demand local depletion for the relief of the local disease. You must not allow yourselves to be beguiled even now by this simulation of inflammatory action. The corded pulse tells of irritative action, but not of one which can be removed by depletion, either local or general. Neither would the resort to blisters be a whit more beneficial. Dependent on the disease of the mucous membrane, it will only yield as that subsides. Happy indeed were it for the patient if it were certain then to disappear. Too often the inflammation results not only in effusion into the cellular tissue, but also in suppuration, either in that tissue or in some of the glands, leaving one of the results of which we shall speak when we come to treat of the sequelæ. This acute glandular enlargement and effusion will yield more kindly to the continuation of poultices and injections than to any other plan. Iodine and the iodide of potassium have been employed both locally and internally, but I believe without any well attested advantage.

The treatment of those cases in which the eruption appears imperfectly developed from the first, or when, after having been well marked, it recedes, or where the force of the attack falls upon the throat, causing great tumefaction and difficulty of deglutition, must be very energetic. In all these cases, this condition evidently results from the overwhelming influence of the poison upon the nervous system. Whether convulsions, or restlessness, or stupor, complicate the case, or mere languor and exhaustion, all are but varying phases of one condition, and that, a condition which is to be removed by appropriate stimulation; and it is in these cases that the capsicum is productive of the happiest results. It was, I confess to you, with great reluctance, I was first prevailed on to resort to a remedy apparently so little appropriate to the treatment of a disease in which the rapid circulation and heated surface seemed rather to call for remedies which should produce a refrigerant impression; and to force a harsh irritating liquid into a throat already inflamed, was, I thought, little short of a refinement of cruelty. The entire failure of the cooling treatment in such cases, led me to test the opposite course, and I can recommend it with entire confidence. Weak animal broths freely charged with capsicum, may be given with great safety, even to the youngest infants; and though it may not—indeed it rarely does—so far rouse the energies of the system as to cause the full development of the eruptive features of the disease, it will so far excite the vital forces as to carry the patient safely through the regular period. Should there be much local disease, it will derive great benefit from the passage of the broth over it. I have often administered this simple infusion when the stomach rejected the broth, or when I desired to maintain a more constant local impression. The common formula of a teaspoonful of powdered capsicum, the same quantity of common table salt, a large spoonful of vinegar, and a half-pint of boiling water, is an exceedingly good one. Of this, a teaspoonful may be given every hour or two to a child of five years old and upwards, followed by a small portion of broth, or even wine-whey. Brandy and quinine have not the same beneficial influence in these cases that they exert over those we shall consider hereafter. They oppress the nervous centres instead of relieving the load. Where there is great restlessness I have derived advantage from moderate doses of morphia, given in the aqua acetatis ammoniæ, or from the Dover's powder. Wine-whey having more tendency to promote the determination to the skin, may be given in every case with safety, where the quinine and brandy would be inappropriate. The resort to hot and stimulating baths and irritative frictions, and the revulsive treatment generally, which would naturally suggest itself, has never been followed by any advantage so far as I have had opportunity for observation, and the internal use of carbonate of ammonia has also disappointed my expectation.

Under the plan of treatment thus indicated, I have found the cases in which the eruption was irregular, defective, or even entirely absent, yield quite as large a percentage of recovery as those which are accompanied with the most intense degree of cutaneous inflammation. It is worthy of remark that such cases are less liable to be followed by dropsical infiltration of the cellular tissue, though the convalescence is more tedious and imperfect.

(To be continued.)

